# Using low-cost drones to map malaria vector habitats

**DOI:** 10.1186/s13071-017-1973-3

**Published:** 2017-01-14

**Authors:** Andy Hardy, Makame Makame, Dónall Cross, Silas Majambere, Mwinyi Msellem

**Affiliations:** 1 Department of Geography and Earth Sciences, Aberystwyth University, Aberystwyth, UK; 2 Zanzibar Malaria Elimination Programme, Zanzibar Ministry of Health, Stone Town, Zanzibar United Republic of Tanzania; 3 Institute of Biological, Environmental and Rural Sciences, Aberystwyth University, Aberystwyth, UK; 4 Innovative Vector Control Consortium, Liverpool School of Tropical Medicine, Liverpool, UK

**Keywords:** Malaria, Drones, Earth observation, Malaria vector habitats, Larval source management

## Abstract

**Background:**

There is a growing awareness that if we are to achieve the ambitious goal of malaria elimination, we must compliment indoor-based vector control interventions (such as bednets and indoor spraying) with outdoor-based interventions such as larval source management (LSM). The effectiveness of LSM is limited by our capacity to identify and map mosquito aquatic habitats. This study provides a proof of concept for the use of a low-cost (< $1000) drone (DJI Phantom) for mapping water bodies in seven sites across Zanzibar including natural water bodies, irrigated and non-irrigated rice paddies, peri-urban and urban locations.

**Results:**

With flying times of less than 30 min for each site, high-resolution (7 cm) georeferenced images were successfully generated for each of the seven sites, covering areas up to 30 ha. Water bodies were readily identifiable in the imagery, as well as ancillary information for planning LSM activities (access routes to water bodies by road and foot) and public health management (e.g. identification of drinking water sources, mapping individual households and the nature of their construction).

**Conclusion:**

The drone-based surveys carried out in this study provide a low-cost and flexible solution to mapping water bodies for operational dissemination of LSM initiatives in mosquito vector-borne disease elimination campaigns. Generated orthomosaics can also be used to provide vital information for other public health planning activities.

**Electronic supplementary material:**

The online version of this article (doi:10.1186/s13071-017-1973-3) contains supplementary material, which is available to authorized users.

## Background

The widespread use of long-lasting insecticide-treated bed nets and indoor residual house spraying has helped to supress malaria transmission across sub-Saharan Africa by targeting vector mosquitoes with a preference for feeding and resting indoors, such as *Anopheles gambiae* (*s.s*.) [1, 2]. For instance, the widespread use of indoor-based interventions in Zanzibar has led to a reduction in malaria prevalence from 40% in 2005 to between 0.2 and 0.5% in 2011/12 [3, 4].

However, these interventions have limited effect for species that show a tendency for feeding and resting outdoors, such as *An. arabiensis*, which are now beginning to dominate transmission throughout sub-Saharan Africa [5–8]. Of greater concern, a growing body of evidence has identified the emergence of pyrethroid resistance in key vector species making them less susceptible to indoor-based interventions that mainly rely on this class of insecticide [5, 9, 10]. As such, it is becoming more apparent that if we are to achieve the ambitious goal of eliminating malaria, it will be necessary to complement indoor interventions with, among others (e.g. mass drug administration), outdoor-based larval source management (LSM) interventions such as larviciding and environmental management of mosquito larval habitats to reduce residual mosquito populations, thereby breaking the malaria transmission cycle [11–20].

The effective implementation of larviciding techniques relies upon our capacity to target interventions at productive mosquito aquatic habitats [5, 21]. Surveys of water bodies over large areas are not feasible from the ground due to the dynamic nature of water bodies; however, there is a body of literature demonstrating the use of earth observation satellites for the detection of malarial mosquito vector habitats. Several studies have successfully used medium-resolution imagery (5–30 m pixels) from systems such as Landsat and SPOT to map water bodies or land cover types associated with malaria transmission [22–29]. Although these types of imagery can provide broad-scale characterisations of surface water they do not offer the necessary resolution for targeting individual water bodies for use in LSM campaigns, nor can they offer support in terms of planning logistics important to the operational implementation of LSM such as the identification of access routes.

Very-high resolution imagery (<5 m pixels) from satellite systems such as IKONOS, QuickBird, WorldView, GeoEye and Pleiades has been used for the detection of individual water bodies (potential vector mosquito aquatic habitats) at a community scale [30–33]. This offers great potential for use in targeting LSM initiatives; however, optical satellite imagery relies on clear-sky conditions that occur infrequently for many regions burdened by malaria. Coupled with infrequent revisit periods, as well as the relatively high cost, this limits the operational use of satellite optical remote sensing [34–36]. Habitat survey teams would need to wait until clear-sky conditions occurred, coinciding with the timing of the satellite overpass, meaning that field teams (that disseminate larvicide and/or undertake environmental management) would need to remain on standby and, in fact, whole seasons may pass without a useable image being available. For instance, a review of the Landsat and Sentinel-2 archives demonstrates that out of the 81 scenes that were centred over Unguja, Zanzibar, since December 2015, no images were completely cloud free; just two images had less than 5% cloud cover and could have been considered useable. Radar systems are not reliant on clear-sky conditions, offering an exciting alternative to optical imagery [34] but this approach is limited by a coarser spatial resolution and the lack of contextual information that is provided by visual analysis of optical imagery.

Unlike high spatial resolution imagery from manned aircraft and satellites that tends to be expensive to acquire as well as being less flexible to operate, drone technology (also known as Unmanned Aerial Vehicles or Systems) offers the potential to obtain very high resolution imagery at a relatively low cost. As well as the ability to identify water bodies and potential habitats [36], drone-based imagery also has the potential to provide ancillary information for planning of logistics: i.e. location and nature of homes, access points/routes to water bodies to direct field teams. Additionally, imagery can be used to establish and monitor links between environmental factors and disease transmission, such as changes in land cover and the emergence of new vector habitats [37, 38]. One of the greatest advantages of drone systems is their flexibility. Although drones cannot be flown in the rain, they are not reliant on clear sky conditions (as they are flown at low altitudes, below clouds, unlike optical satellites) and the timing of satellite overpasses and they can therefore be flown at times convenient for field teams, making them an ideal tool for supporting LSM initiatives [36].

The use of drones for tackling the global burden of malaria is in its infancy. Investigations have been made into the potential for drone technology to disseminate larvicide using heavy lift and long endurance drones [39]. Fornace et al. [36] carried out a case study exploring the use of drone technology for mapping environmental risks for zoonotic malaria associated with changes in land use but did not attempt to use the imagery to map aquatic vector habitats themselves. To date, there have been no reported attempts to use drone technology to identify water bodies as a target for LSM interventions. The aim of this study is to provide a proof of concept for the use of low-cost (< $1000) drones for mapping water bodies as targets for LSM.

## Methods

### Study site

**Fig. 1:**
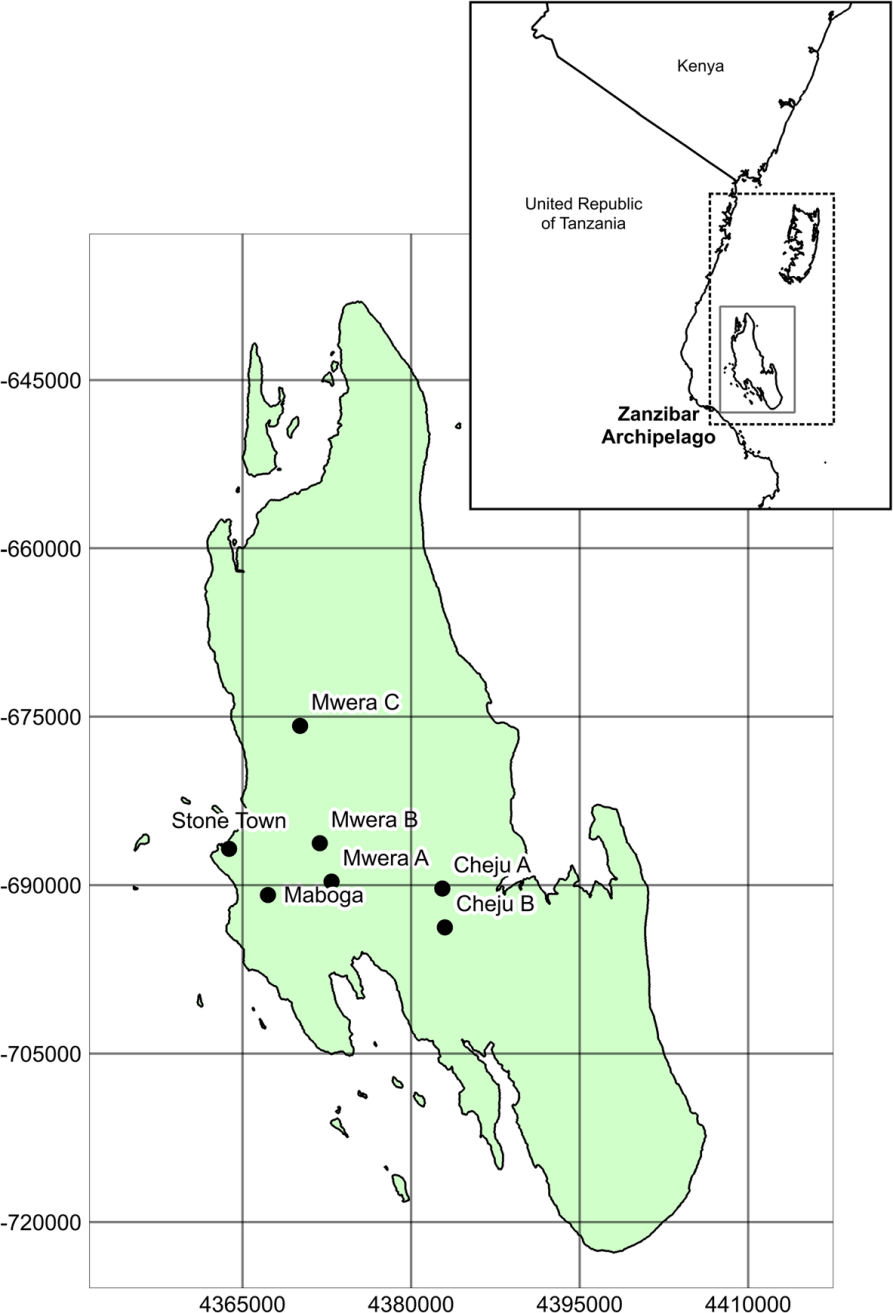
Map showing the location of Unguja within the Zanzibar Archipelago and sites surveyed in the study

This study is located on the island of Unguja, the main island of the Zanzibar archipelago (Fig. 1). Unguja receives between 1000 and 2250 mm of rainfall per year. Rainfall is strongly seasonal, typically with dry and hot weather during January and February, heavy rains from March to May, a dry season during June to September and light rains during October to December [40].

Unguja is characterised by a karstic geology, largely comprising coralline limestone with high soil infiltration rates occurring in most areas apart from doline areas where fine-grained sediment supports shallow water bodies and cultivation [41]. The land cover is largely vegetated comprising secondary forest, mangrove swamps, and degraded fallow bush. Agriculture is mainly dominated by root crops, vegetables and both rain-fed and irrigated rice plantations [41, 42]. There is one main urban settlement in Zanzibar, Stone Town, which accounts for approximately 20% of the total population of the archipelago.

The Zanzibar Malaria Elimination Programme (ZAMEP: Zanzibar Ministry of Health) plans to undertake LSM initiatives as part of the elimination phase of their programme. Unpublished pilot work conducted by ZAMEP disseminated methoprene (an insect growth regulator) in habitats at two island locations (Uzi and Kisiwa Panza). Before doing so, ZAMEP field entomological teams, in conjunction with local communities, produced an inventory of water bodies within the pilot study areas. However, this was a time-consuming process and often resulted in inconsistent or inaccurate water body maps, in some cases leading to water bodies being missed out of the LSM trial (ZAMEP, 2016, pers comm., 11 June). Household level surveillance of malaria cases by ZAMEP offers the ability to map transmission hotspots, helping to target LSM initiatives, but as yet they do not have a reliable method for mapping water bodies and potential vector habitats within these hotspot areas of transmission.

### Drone surveying and processing

**Fig. 2:**
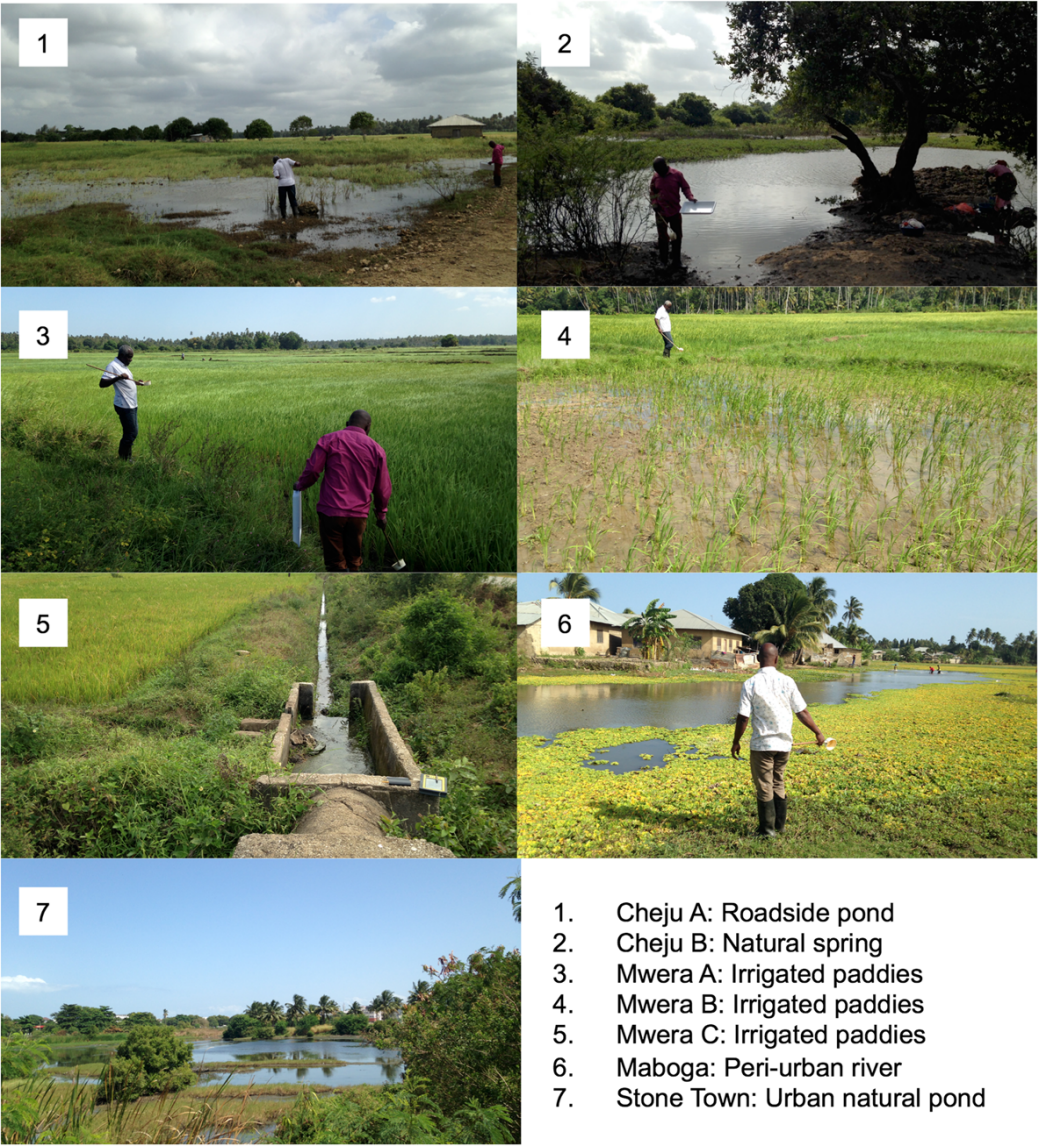
Photos of the seven sites surveyed using the low-cost drone system representing the land cover types: (i) Seasonally wetted rice paddy (Cheju A); (ii) irrigated rice paddy (Mwera A, B and C); (iii) natural spring-fed pond (Cheju B); (iv) peri-urban suburb of Stone Town (Maboga) and; (v) urban (Stone Town)

In June 2016 drone surveys were carried out at seven sites (Fig. 1) which were broadly representative of different land cover types occurring across Zanzibar (Fig. 2): (i) seasonally wetted rice paddy (Cheju A); (ii) irrigated rice paddy (Mwera A, B and C); (iii) natural spring-fed pond (Cheju B); (iv) peri-urban suburb of Stone Town (Maboga); and (v) urban (Stone Town). Cheju and Mwera are situated in the Central administrative district that has higher rates of malaria positivity (July-September 2015: 4.5%) compared to rates for the rest of the Zanzibar archipelago, including the Urban district in which Stone Town and Maboga are situated (July-September 2015: 2.5%) [43].

The drone surveys were carried out using a DJI Phantom 3 (DJI, Shenzhen, China: http://www.dji.com) quadcopter system fitted with a DJI 4K Edition camera (Sony Exmor R Model IMX117: 7.81 mm CMOS sensor, 4000 × 3000 12 Megapixel) with an f/2.8 lens and a 94° field of view. This system, together with computer tablet for operating the quadcopter, is widely available for less than $1000. At each site, the drone was flown to an altitude of approximately 100 m. This altitude provided images with an approximate ground footprint of 130 × 180 m with a spatial resolution of 7 cm.

**Fig. 3:**
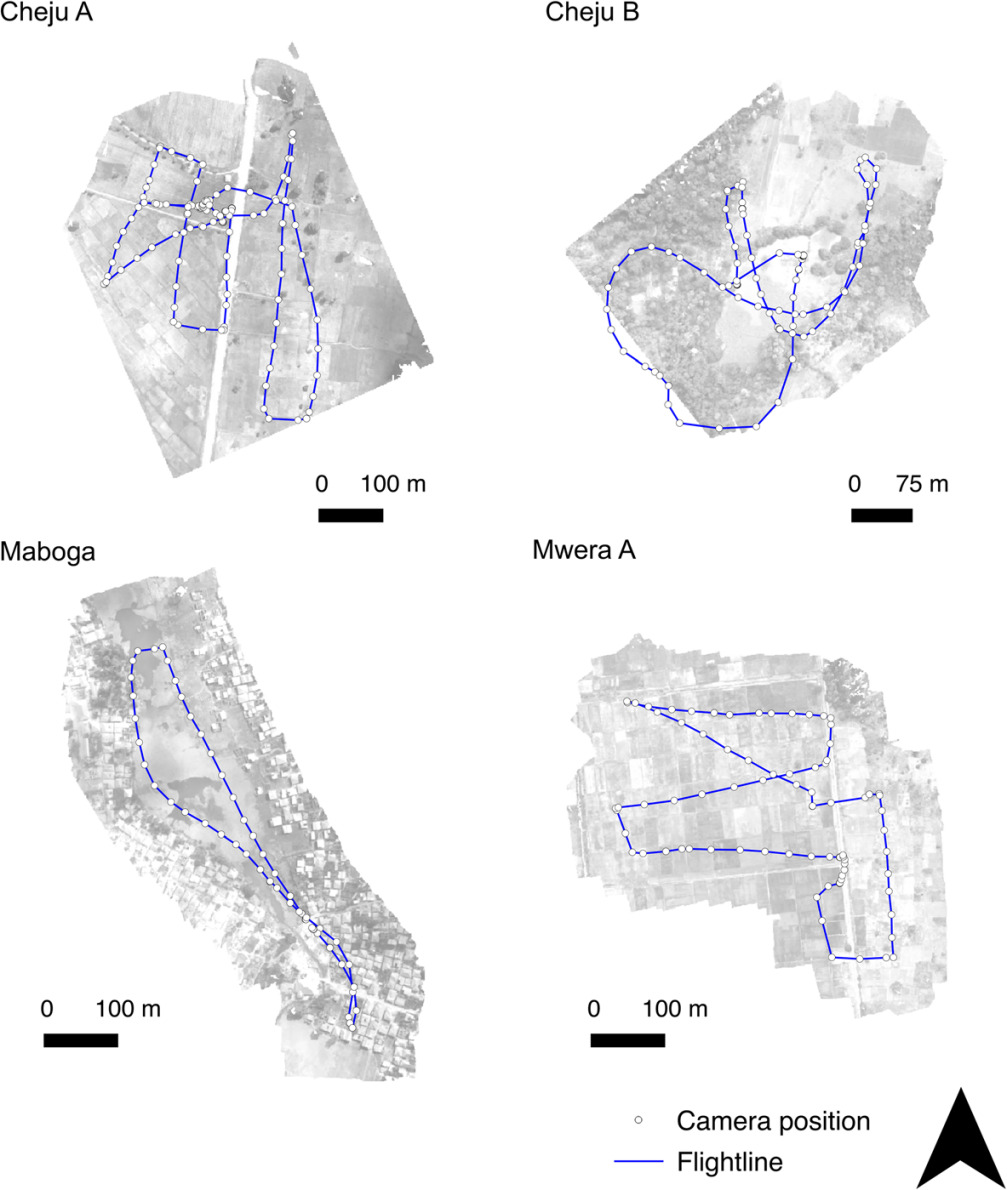
Locations of flight lines and subsequent central camera positions for each drone photo for sites at Cheju A, Cheju B, Maboga and Mwera A

**Fig. 4:**
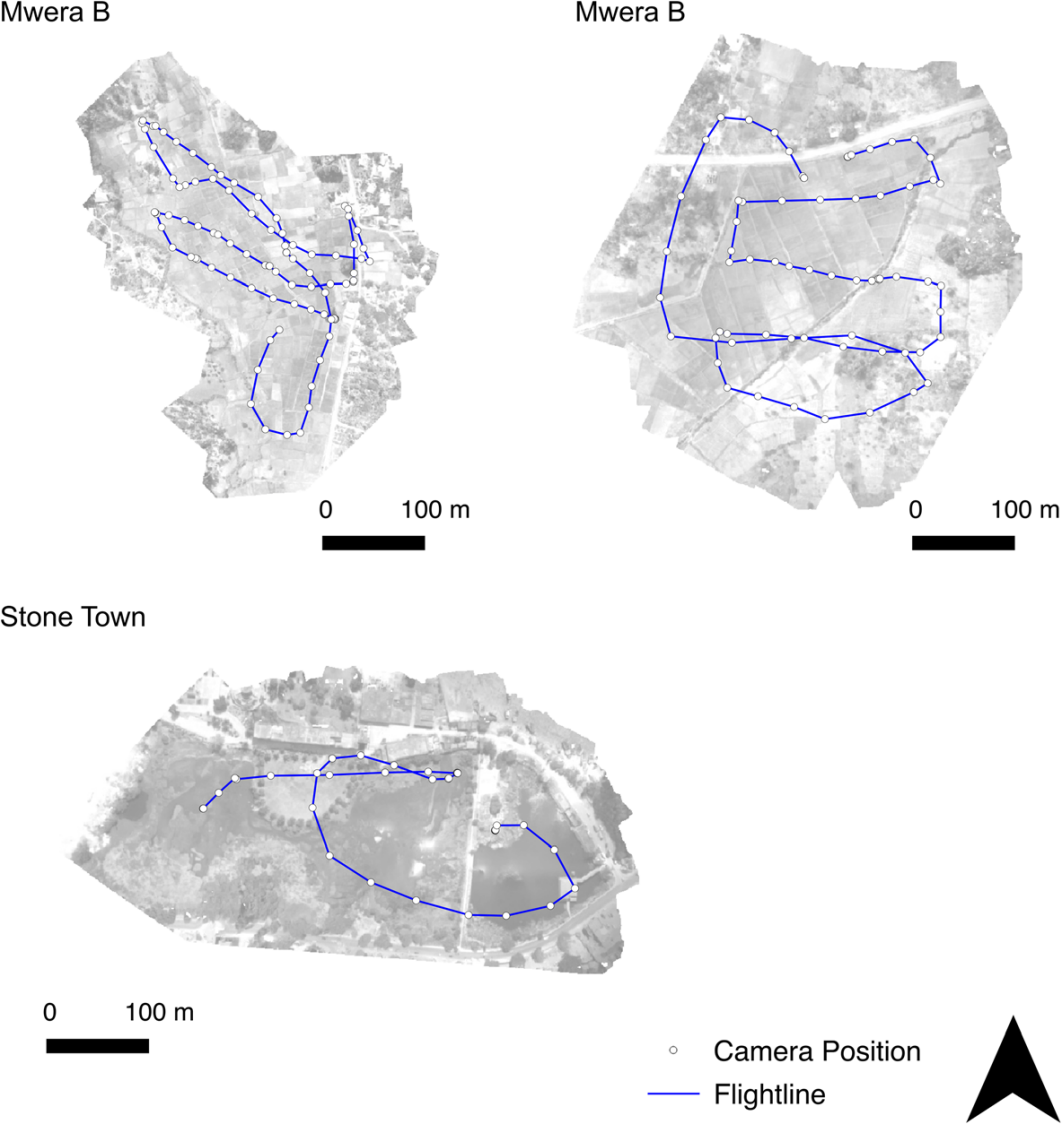
Locations of flight lines and subsequent central camera positions for each drone photo for sites at Mwera B, Mwera C and Stone Town

The camera was programmed to take a photograph every five seconds ensuring an overlap of between 60 and 70% between each pair of neighbouring images. The quadcopter cannot be flown in the rain but can operate safely in wind: wind gusts of between 25 and 32 km/h were experienced during this study, presenting no challenges for the flight of the quadcopter or the resulting imagery. For each flight the cloud cover was < 20%. Flight lines were determined in the field with imagery being flown in strips through reference to the live footage on the tablet, ensuring sufficient coverage of the site and sufficient overlap (60–70%) necessary for generating an orthophoto (see Figs. 3 and 4 for flight lines and camera positions). Flight lines were followed manually; however, waymarkers can be recorded during the manual flight and automatically reflown at a later date to aid repeatability.

The airspace regulations as defined by the Tanzania Civil Aviation Authority were followed (maintaining line of site with drone, operating at altitudes < 120 m, not flying over national parks or airfields). Additional safety measures are provided by the DJI operating software that detects no-fly zones and alerts the operator if a no-fly zone is detected. Each site was surveyed in a single flight without a change of battery resulting in flying times of less than 20 min. The specific flight time per site can be found in Table 1.

**Table 1:** Summary of drone surveys and costs for field survey per site. Cost estimates based on one 4 × 4 vehicle plus driver and fuel, two technical field entomological surveyors plus surveying equipment, community assistant and managerial coordination

Name	Description	Flight time (min)	Total area surveyed (ha)	Cost for field survey team
Cheju_A	Roadside borrow pit	16	28.6	$70
Cheju_B	Natural waterbody	12	16.0	$34
Maboga	Slow moving perennial river	7	18.6	$40
Mwera_A	Irrigated paddy	7	29.8	$64
Mwera_B	Irrigated paddy	10	22.6	$48
Mwera_C	Irrigated paddy	7	18.8	$40
Stone Town	Natural pond close to roadside and coast	6	14.8	$32

**Fig. 5:**
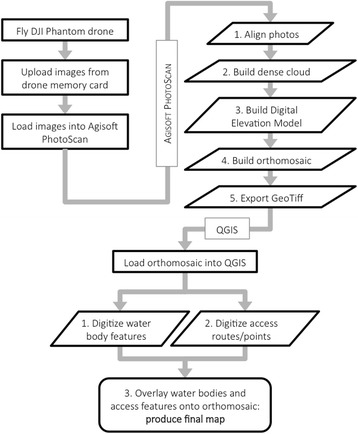
Workflow diagram summarising the image processing steps in Agisoft PhotoScan and QGIS

A summary of the image processing steps can be found in Fig. 5. The resulting imagery was imported into AgisSoft Photoscan Pro (https://www.agisoft.com) and processed to extract an orthomosaic (a georeferenced mosaic of overlapping photographs or images which includes correction for topographic distortions) for each site following a standard procedure: (i) align photos (precision: high; alignment: reference); (ii) build dense point cloud (quality: high; depth filtering: moderate); (iii) build digital elevation model (DEM) (7 cm pixel size; interpolation: extrapolated; all point classes to generate digital surface model); (iv) build orthomosaic (input surface: DEM; blending mode: mosaic). The position of the drone at the time of image capture for each photo is recorded automatically by the on-board GPS; as such, the orthomosaic can be georeferenced (i.e. placing the mosaicked image into a map coordinate system) automatically by the software without the need for reference images, maps or Ground Control Points (GCPs: an accurately surveyed reference point or feature that can be used to geo-reference a subsequent image or improve its locational accuracy).

Once processed, the resulting orthomosaic was imported into QGIS (www.qgis.org) where manual interpretation was used to delineate water body location and size. Although time-consuming (each orthomosaic taking between 30 min and two hours to manually interpret), this ensures a high level of accuracy, as well as enabling the interpretation of complex contextual information (e.g. distinguishing water bodies with high suspended sediment from surrounding bare earth, identification of surface water due to the relatively smooth texture, identifying waterbodies masked by shadow or aquatic vegetation). Delineated water bodies, together with other potential useful information identified in the image analysis are overlaid onto the orthomosaic in QGIS and used to generate printed maps for ZAMEP field entomological survey teams.

### Entomological survey

Alongside the drone survey a simple entomological survey was conducted at each site to determine the presence of anopheline/culicine larvae and pupae as well as environmental characteristics such as evidence of predators, cattle access, presence of aquatic vegetation, including algae, degree of turbidity and shade, and water body dimensions. The survey design followed ZAMEP standard operating procedures in which the fringes of water bodies identified at the survey site are walked with larval samples being taken using a standard 350 ml dipper following a purposive dipping strategy [17].

## Results and discussion

**Fig. 6:**
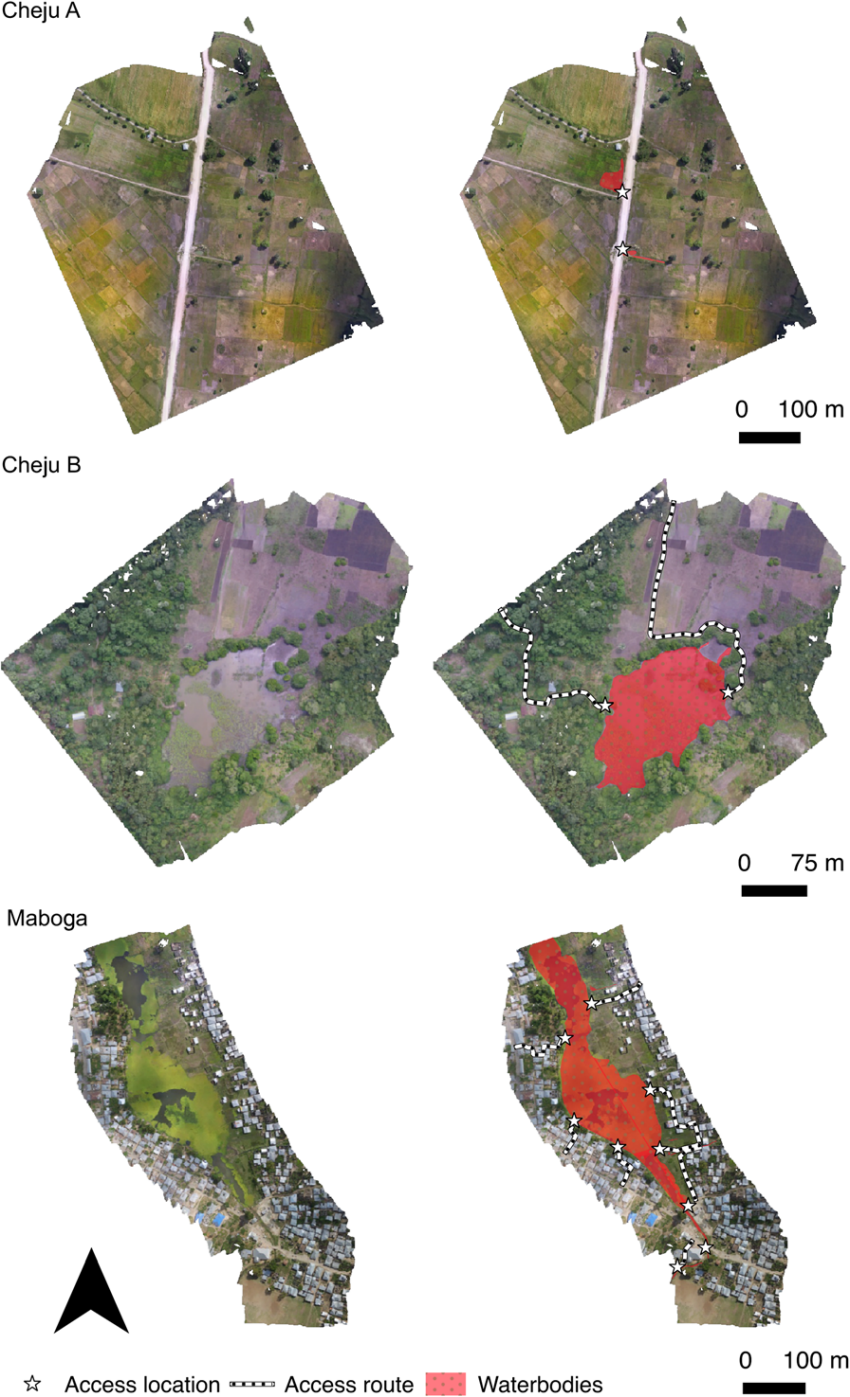
Orthophotos for sites at Cheju A, Cheju B and Maboga, including mapped water bodies, access routes and access points

**Fig. 7:**
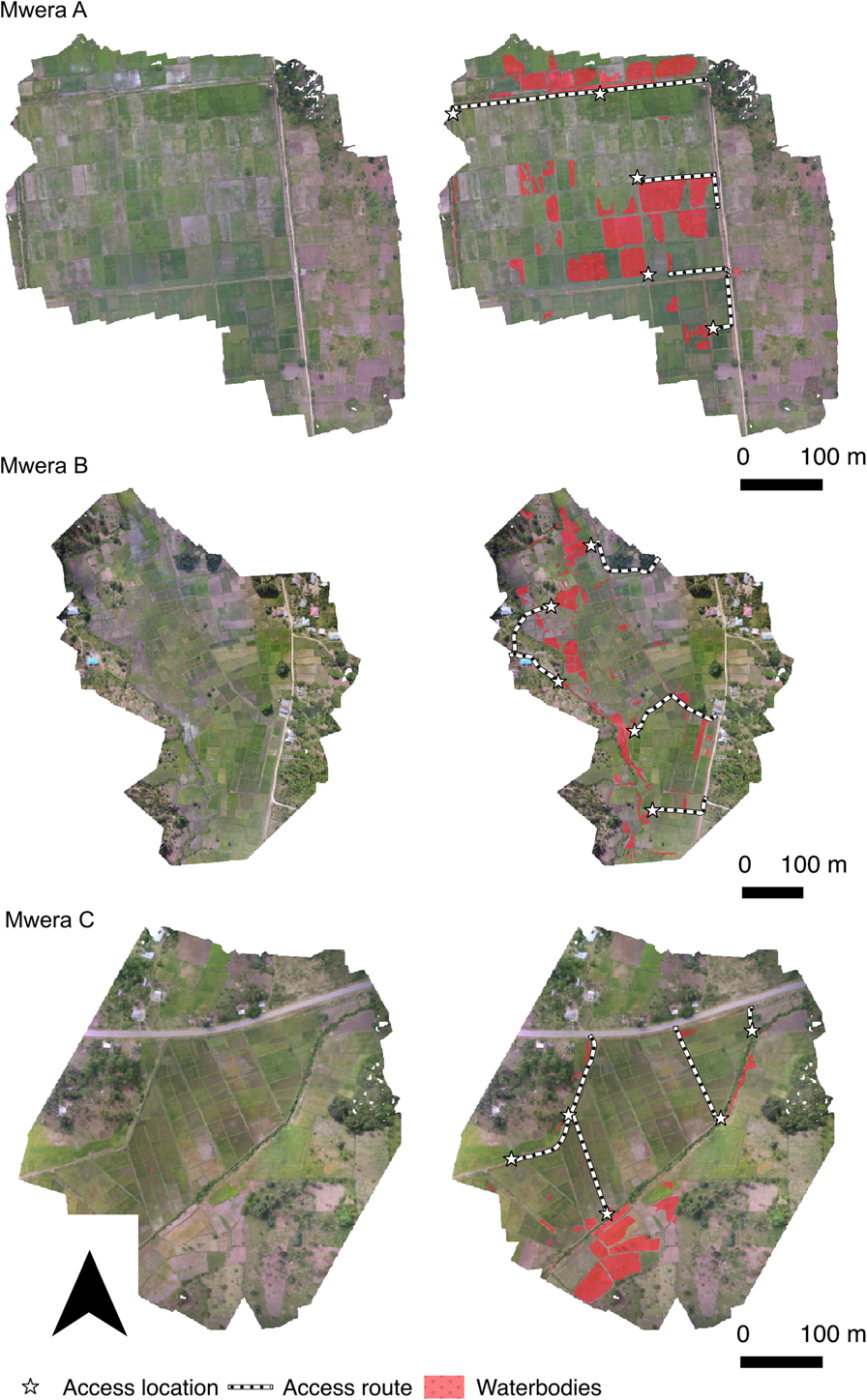
Orthophotos for sites at Mwera A, Mwera B and Mwera C, including mapped water bodies, access routes and access points

**Fig. 8:**
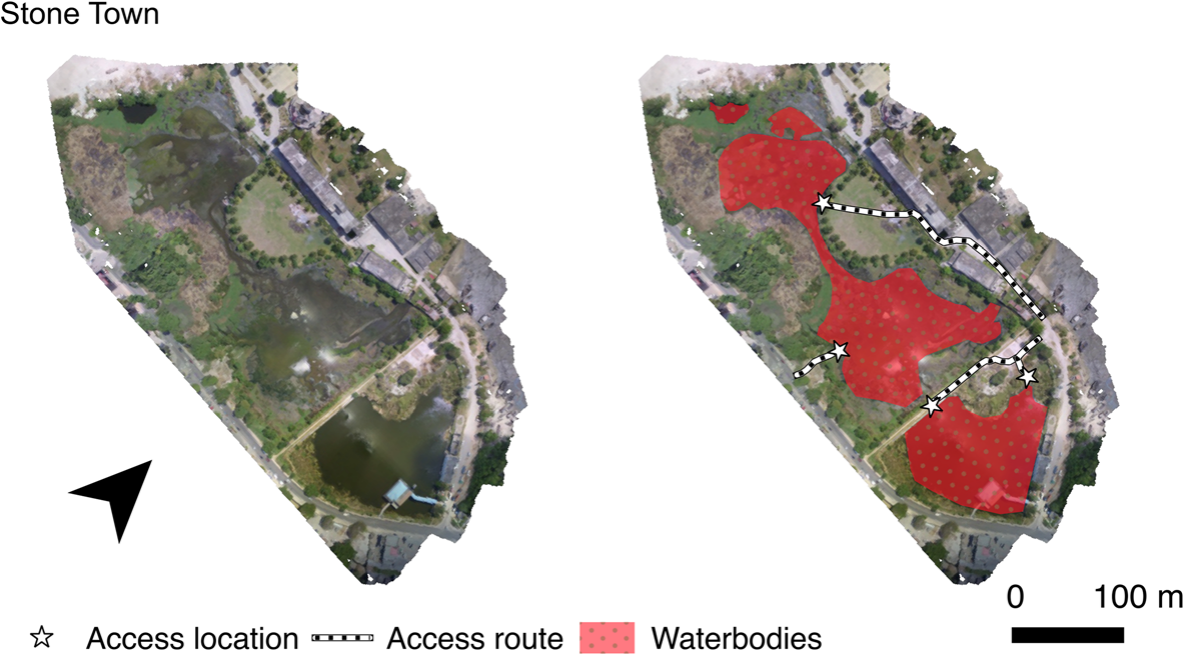
Orthophotos for sites at Stone Town, including mapped water bodies, access routes and access points

**Fig. 9:**
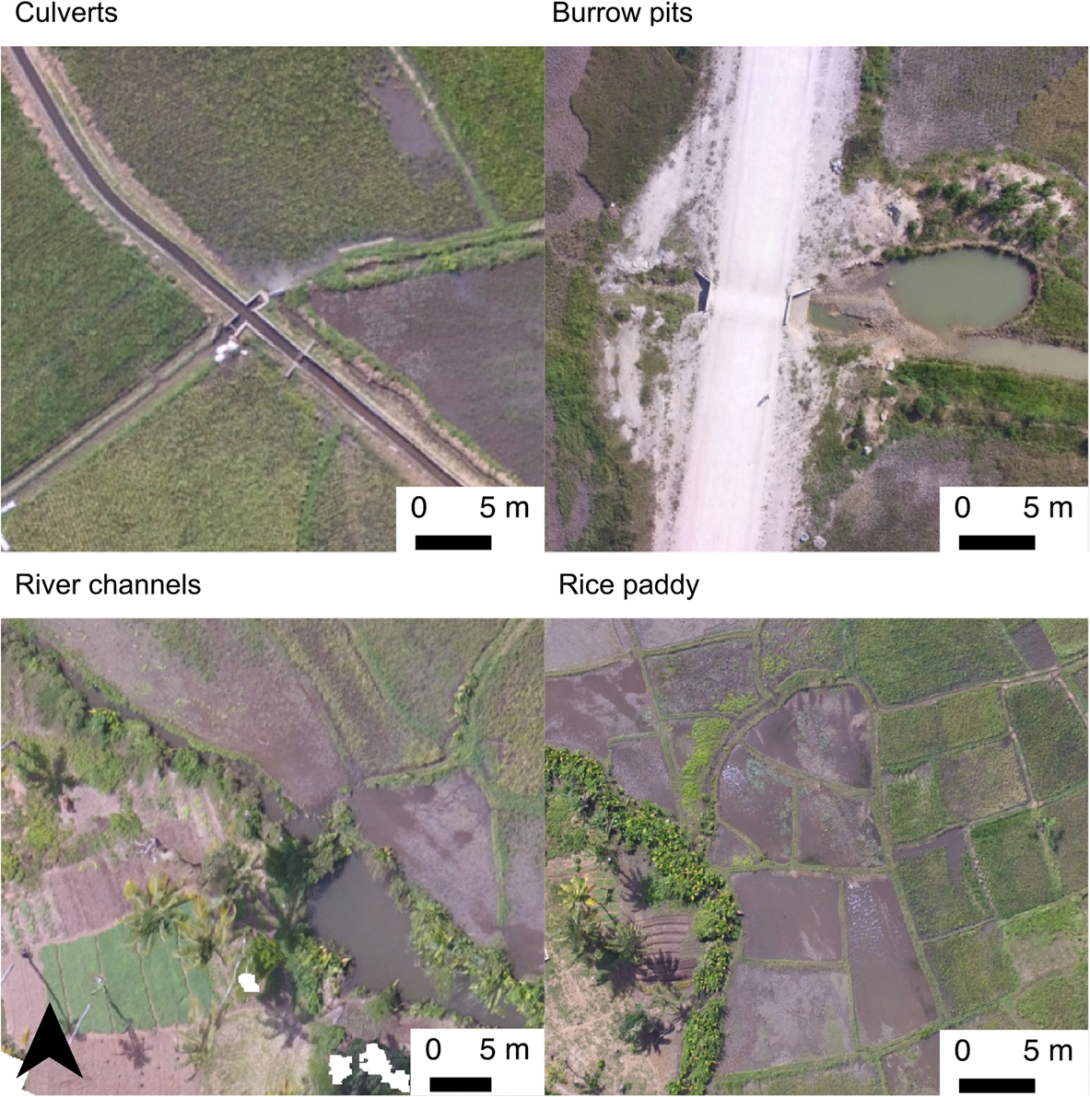
Example water body types identified in the orthophotos including those associated with culverts, roadside borrow pits, river channels and rice paddies

Overlapping photos collected in the drone survey were successfully processed to produce orthomosaics for each of the seven sites (Figs. 6, 7 and 8). Processing of the imagery for each site took approximately two-three hours in Photoscan Pro using a standard laptop (16 GB RAM). Each site was surveyed in a single flight taking less than 30 min including the time taken to set the drone up, fly the drone and pack the equipment away, including sites up to 30 ha in extent (Table 1). This represents a scale and resolution that is sufficient for operational surveying of water body habitats at the community scale including water bodies amongst rice paddies, culverts and associated water bodies, river channels and streams, natural water bodies such as springs but also smaller pools of water associated with borrow pits at the side of roads (Fig. 9).

All of the habitats identified by the field entomological team were correctly identified in the relevant orthomosaic (Table 2). Furthermore, the drone imagery was able to identify a large number of potential habitats not identified by the field team; in most instances, almost half the water bodies identified in the drone orthomosaics were missed by the field survey. These water bodies were not identified on the ground largely because they were obscured from immediate view (i.e. within/behind dense vegetation canopies, behind housing and other structures, too small to be detectable at a distance from the ground), highlighting the need to undertake synoptic surveys of water bodies, using remote sensing technologies such as drones, to generate inventories of potential mosquito aquatic habitats. The cost for surveying all seven sites from the ground was approximately US$320 compared to the cost of US$1000 for the drone surveying equipment. Despite the initial high cost, the drone survey was much more accurate than ground-based field surveys and covered a greater area in a much shorter length of time. More significantly, the cost of the equipment would be justified after repeating the same survey two or three times, thereby representing a longer-term strategy for providing more cost-effective surveys of potential malarial mosquito habitats.

**Table 2:** Comparison of the number and extent of water bodies identified using field observations and through analysis of drone imagery

Name	No. water bodies identified		Surface water extent (m^2^)		Mean water body size (m^2^)
	Field	Drone	Field	Drone	
Cheju_A	1	4	1342.9	1626.5	406.6
Cheju_B	1	3	18,275.7	18,443.2	6147.7
Maboga	6	11	35,524.35	35,780.3	3252.8
Mwera_A	30	87	14,933.1	30,000.5	344.8
Mwera_B	49	143	2350.4	13,888.9	97.1
Mwera_C	28	64	816	7743.1	121
Stone Town	2	4	34,580.4	36,104.6	9026.1

Irrigated rice paddy areas were characterised by a large number of relatively small water bodies whereas naturally occurring water bodies tended to be large but infrequent (Table 1). Those naturally occurring water bodies sampled in peri-urban and urban sites contained no anopheline larvae (Table 3); only *Culex* larvae were found to be present in the peri-urban site at Maboga, sampled at the fringes of a large water body associated with a slow-moving perennial river. By contrast, water bodies located within irrigated rice paddies were shown to be positive for anopheline larvae, particularly in Mwera A and B with over 100 early instar stage larvae being found in 40 dips (Table 3). Additionally, the naturally occurring water body in a rural location (Cheju B) was also sampled positive for anopheline larvae (Table 3).

**Table 3:** Summary of entomological survey and environmental characterisation. Evidence of cattle visiting the site (yes or no); presence of algae (0: absent, 1: < 25% coverage, 2: 25–50%, 3: 50–75%, 4: > 75%); presence of aquatic vegetation (0: absent, 1: < 25% coverage, 2: 25–50%, 3: 50–75%, 4: > 75%); degree of shading (0: no shade, 1: < 25% coverage, 2: 25–50%, 3: 50–75%, 4: > 75%); predator prevalence (0: no predators, 1: low, 2: moderate, 3: abundant); counts of: early instar stage anopheline larvae, late stage anopheline larvae, mosquito pupae, culicine larvae, number of dips taken

Name	Cattle	Algae	Aq. Veg.	Turb.	Shade	Pred.	An. early	An. late	Pupae	Culicine	Dips
Cheju_A	Y	1	4	1	0	2	0	0	0	0	40
Cheju_B	Y	0	2	3	1	2	3	2	0	1	40
Maboga	Y	2	4	3	0	3	0	0	0	83	20
Mwera_A	N	0	4	0	1	0	106	4	0	1	40
Mwera_B	N	0	4	0	1	1	103	16	1	15	40
Mwera_C	N	0	4	0	1	0	0	4	0	0	20
Stone Town	N	1	3	2	2	3	0	0	0	0	20

*Abbreviations*: *Aq. veg.* aquatic vegetation, *Turb.* tubidity, *Pred.* predators, *An.* early, early instar stage anopheline larvae, *An late* late instar stage anopheline larvae

### Limitations

As is the case with all optical imagery, surveys cannot be made of water bodes situated under dense tree canopies. However, in the application of malaria disease control, evidence suggests that these water bodies tend not to be productive anopheline habitats in sub-Saharan Africa with species favouring open, sun-lit bodies of water [32, 44–47]. Nevertheless, this limitation must be acknowledged in any subsequent survey work. Additionally, shadows created by tall trees and buildings can obscure potential water bodies. This factor can be reduced by avoiding drone surveys when sun angles are low (i.e. early morning/evening).

To increase the positional accuracy of the generated orthomosaic, ground control points (GCPs) or reference markers can be incorporated into the processing stream. However, as the position of the drone photos were recorded by the onboard GPS the collection of GCPs was not necessary to produce an orthomosaic, although this would have increased the positional accuracy of the orthomosaic. Additionally, input GCPs must be very accurate (cm level) requiring use of GNSS (Global Navigation Satellite System) differential GPS system that was not available at the study site. With the addition of highly accurate GCPs a digital elevation model (DEM) can be extracted from the imagery. Although DEMs can provide a great deal of information regarding the likely location of water bodies in areas where hydrology is mainly a function of topography, this is not the case in Zanzibar due to the islands’ geology [41].

The identification of water bodies in the resulting orthomosaic can sometimes be difficult, particularly when water bodies with high suspended sediment are the same colour as bare soil. This complication was overcome by manually interpreting texture patterns; water bodies tend to be much smoother than bare ground or other land cover types. In this study, field observations were used to validate the image analysis, particularly where sites had abundant floating vegetation such as lily pads. Field validation can be performed by field entomological teams that collect baseline data for larviciding/environmental management. Future work might consider the use of drone-mounted optical systems operating beyond the visible range of the spectrum, particularly in the near-infrared and shortwave infrared regions of spectrum where water bodies are spectrally distinct [25]. Efforts could also be made in the future for automatic detection of water bodies using drone imagery (such as Casado et al. [48]); the calibration/training of such models, however, is relatively time-consuming and therefore manual interpretation and delineation of water bodies, as demonstrated to be successful in this study, represents a more operationally valid option for malaria managers.

Some gaps occurred in the generated orthophotos where insufficient image overlap occurs. This tended to occur towards the edge of the image, outside the immediate area of interest, and therefore did not have a large impact on the usefulness of the imagery. Nevertheless, operators should ensure sufficient overlap in acquired photos that can be achieved with relatively low flight speeds and a short duration between image capture (five second repetition time was used in this study). To further explore the optimum drone operating protocol future studies may consider flying the drone at a range of flight speeds and altitudes, as well as other factors such as varying angle of image capture and camera settings (e.g. ISO sensitivity, shutter speed, image format, white balance). Guidelines and protocols for flying drones in most international airspace stipulate the requirement to always keep the system within line of sight. As such, the drone operator was limited to altitudes of < 120 m. Nevertheless, this represented a flying height that optimised the coverage of each image on the ground (approximately 130 × 180 m) while maintaining a high spatial resolution (7 cm).

Most drone systems, including the model used in this study, cannot be flown during rainfall conditions. The drone surveys undertaken for this study took place at the start of the dry season and no rainfall was experienced, however, the likelihood of success is likely to decrease during the wet season making drone surveys less suitable for operational use during this time of the year (typically March to May). However, it would be preferable to carry out LSM interventions during the dry season during which the number of aquatic habitats are relatively limited, following the dry season refugia concept [49].

The capacity of the field entomological survey was limited due to the relatively small size of the field team. Only two technical entomological surveyors were used to carry out the ground-based survey of potential mosquito aquatic habitats, making it difficult to sufficiently survey the area covered by the drone survey (up to 30 ha). Future studies may consider a more comprehensive assessment of the abilities of ground-based surveying by employing more surveyors. This study used a relatively small number of two surveyors at each site as this is the number typically used by ZAMEP to conduct their entomological surveys. However, in practice, more surveyors may be available in the event of a LSM trial, i.e. those conducting entomological surveys in water bodies as well as the team responsible for disseminating the larvicide/environmental management.

## Conclusions

To successfully eliminate malaria, we need to complement indoor-based vector control interventions with outdoor-based initiatives such as LSM [11–20]. To make LSM feasible, tools are needed to identify water bodies at a community scale to target resources [50]. The methods outlined in this paper proved to be successful in generating orthophotos at a scale and resolution (7 cm resolution up to a 30 ha area) that are suitable for targeting entomological surveys, larviciding or environmental management activities by LSM field teams.

**Fig. 10:**
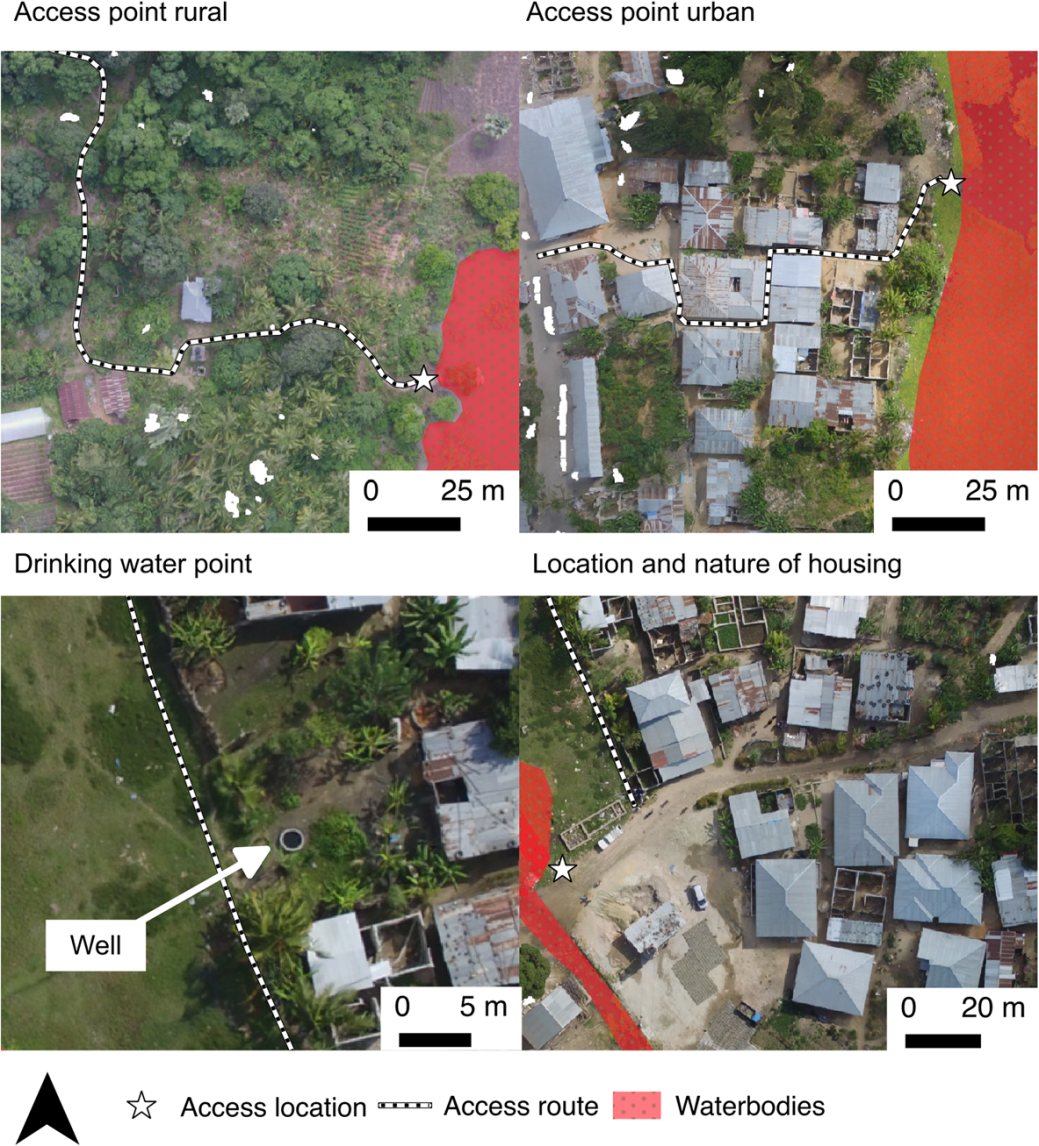
Examples of ancillary information identified in orthophotos to facilitate Larval Source Management and public health activities

As well as providing information regarding the location and extent of water bodies within target areas, analysis of the orthophotos can be used to identify access routes to direct field entomological teams to particular water bodies. Such imagery can also be used to map features such as individual households or outdoor drinking water sources to support other public health initiatives. For instance, mass drug administration schemes or treated bednet dissemination campaigns require accurate information regarding location and number of dwellings. Drone-derived orthophotos, such as those collected in this study, can be used to identify individual properties and even the characteristics of that property, such as its construction (grass roofs, corrugated iron, greenhouse) or size (Fig. 10). Additionally, derived orthophotos can be used to map drinking water sources such as wells that may be necessary in controlling outbreaks of water-borne diseases such as cholera (Fig. 10).

A growing application of drone imagery is in the derivation of DEM products. Specifically, a large number of overlapping photos (as obtained in this study) can be used to generate a DEM using photogrammetric techniques, namely structure from motion [51–54]. The resulting DEM can be used to drive a range of physically-based hydrological models that can be used to predict the location and timing of surface water bodies [55–58]. However, this approach is not applicable to areas like Zanzibar which are limestone-dominated karst environments which promote the underground movement of water and therefore surface topographic controls on water accumulation are limited [41]. Nevertheless, this represents an exciting opportunity for application in other regions burdened by malaria in which topography plays a key role in driving surface water availability, such as the Western Kenyan Highlands [32, 59–63].

The drone-based orthophotos developed in this study provide sufficient resolution to identify water bodies and access points for targeting LSM efforts. Furthermore, the approach and drone system employed gives flexibility to operators so that surveys can be timed with field-based activities as well as providing a low-cost means for carrying out repeated surveys at convenient times. As such, this study demonstrates a clear potential for operational use of such technologies in elimination campaigns using LSM. Although reasonably large areas can be surveyed in short amount of time (30 min flying for a 30 ha area), this approach should be carried out in a targeted fashion, focussed on known malaria transmission hotspots.

## Additional files

Additional file 1: Georeferenced orthoimage for Chuju A. (TIF 116921 kb)
Additional file 2: Georeferenced orthoimage for Cheju B. (TIF 67111 kb)
Additional file 3: Georeferenced orthoimage for Mwera A. (TIF 74943 kb)
Additional file 4: Georeferenced orthoimage for Mwera B. (TIF 114369 kb)
Additional file 5: Georeferenced orthoimage for Mwera C. (TIF 99069 kb)
Additional file 6: Georeferenced orthoimage for Stone Town. (TIF 79772 kb)
Additional file 7: Georeferenced orthoimage for Maboga. (TIF 59641 kb)

## Abbreviations

DEM: Digital elevation model
GCP: Ground control point
GPS: Global positioning system
LSM: Larval source management
Orthomosaic: A georeferenced mosaic of overlapping photographs
ZAMEP: Zanzibar malaria elimination programme
