# Supplementary figures and images for: Using low-cost drones to map malaria vector habitats
Source: Parasit Vectors. 2017 Jan 14;10:29. doi: 10.1186/s13071-017-1973-3 (PMC5237572; doi:10.1186/s13071-017-1973-3)

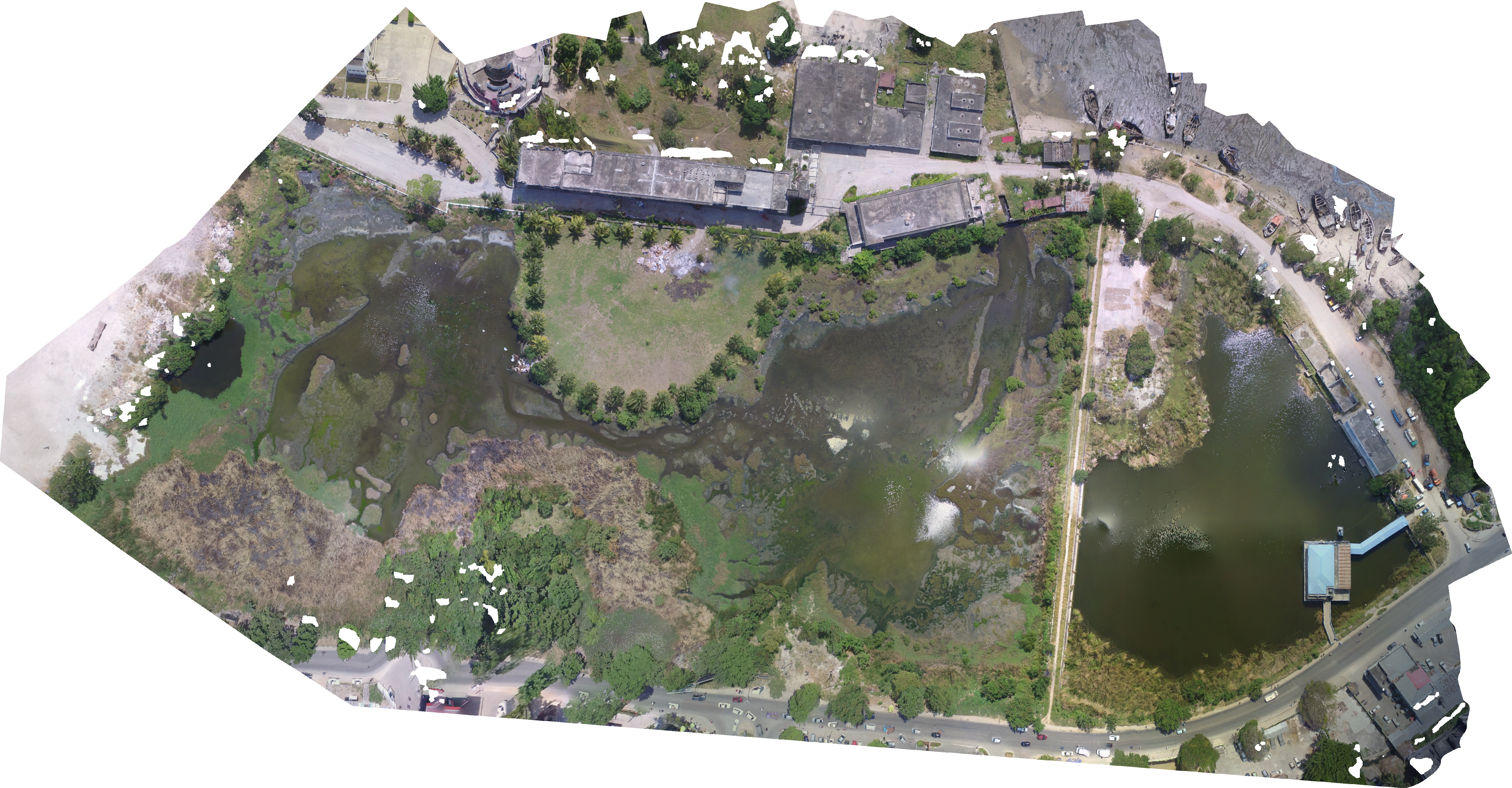

Supplement: Additional file 7: — Georeferenced orthoimage for Maboga. (TIF 59641 kb) [file 13071_2017_1973_MOESM7_ESM.tif]
